# Validation and Evaluation of Selected Organic Pollutants in Shrimp and Seawater Samples from the NW Portuguese Coast

**DOI:** 10.3390/molecules26195774

**Published:** 2021-09-23

**Authors:** Maria Luz Maia, Cristina Delerue-Matos, Conceição Calhau, Valentina Fernandes Domingues

**Affiliations:** 1 REQUIMTE/LAQV-GRAQ, Instituto Superior de Engenharia do Porto, Politécnico do Porto, 4249-015 Porto, Portugal; mariadaluz.maia@gmail.com (M.L.M.); cmm@isep.ipp.pt (C.D.-M.); 2 Center for Research in Health Technologies and Information Systems, 4200-450 Porto, Portugal; ccalhau@nms.unl.pt; 3 Nutrição e Metabolismo, NOVA Medical School, Faculdade de Ciências Médicas, Universidade Nova de Lisboa, 1169-056 Lisboa, Portugal

**Keywords:** endocrine disruptors, aquatic environment, *Palaemon serratus*, *Palaemon varians*, bioaccumulation, biomonitoring

## Abstract

The development of coastal regions has contributed to the intensification of environmental contamination, which can accumulate in aquatic biota, such as shrimps. These crustaceans, besides being delicious and being a good source of nutrients, can also accumulate environmental pollutants. Amongst others, these include organochlorine pesticides (OCPs), organophosphorus pesticides (OPPs), brominated flame retardants (BFRs), polychlorinated biphenyls (PCBs) and synthetic musks (SMs). These pollutants, classified as endocrine disruptors, are related to adverse effects in humans and since one of the major routes of exposition is ingestion, this is a cause for concern regarding their presence in food. The aim of the present study was to quantify the presence of environmental pollutants in shrimp samples and in the water from their habitat along the northwest Portuguese coast. In seawater samples, only two OCPs (lindane and DDD) and one BFR (BTBPE) were detected, and in shrimp samples, one OCP (DDD) and three SMs (HHCB, AHTN and ketone) were found. Bioaccumulation and the risk assessment of dietary exposure of SMs in shrimp samples were investigated. It was observed that all shrimp samples analyzed significantly presented bioaccumulation of the three SMs found. Concentrations of SMs detected in shrimp samples do not present a health risk for the adult Portuguese population.

## 1. Introduction

In the last several decades, rapid commercial, agricultural and industrial development has contributed to increasing environmental pollution. Oceans are a reservoir of pollutants and play an important role in the transport and fate of environmental pollutants. The large seawater volume in oceans makes this matrix an important inventory for pollutants, particularly in coastal regions [1]. Environmental pollution of surface waters poses a threat to the aquatic environment, through the toxic effects on aquatic organisms. The accumulation of pollutants in the ecosystem is also a potential hazard to human health. As a result, protection of water resources and biota is a priority. The European Union (EU) released maximum allowable concentration levels for some pollutants in surface waters [2]. In aquatic environments, hydrophobic pollutants can bioaccumulate though direct uptake from the water or trophic transfer [3]. This creates safety concerns for human consumption of aquatic organisms. Shrimp is one of the most popular crustaceans consumed worldwide and can be a healthy addition to our diet. Shrimp is low in fat and calories, rich in omega-3 fatty acids and a good source of key nutrients, such as iodine, phosphorus, choline, copper, zinc, B-complex vitamins, vitamin A and E and antioxidants, especially astaxanthin [4,5].

Shrimps can accumulate pollutants from their surrounding environment. These pollutants include organochlorine pesticides (OCPs), organophosphorus pesticides (OPPs), brominated flame retardants (BFRs), polychlorinated biphenyls (PCBs) and synthetic musks (SMs). These chemicals are lipophilic endocrine disruptors that are persistent in the environment and can be transferred through the food chain [6,7,8]. These pollutants are a global problem for the environment and human health.

OCPs have been used extensively in agriculture as pest and insect control. The use of OCPs, such as dichlorodiphenyltrichloroethane (DDT), hexachlorobenzene (HCB), aldrin, endrin, chlordane, heptachlor, mirex and toxaphene, have been prohibited in many European, North American and South American countries from the 1970s and 1980s [9,10]. Despite this, DDT and lindane (γ-HCH) are still used in some countries. DDT is used to inhibit mosquito growth to prevent vector-borne diseases such as dengue, leishmaniosis, malaria and Japanese encephalitis and lindane is used to treat head lice in children [11]. 

OPPs are the class of agriculture pesticides most used worldwide. Besides their agricultural use, OPPs also have many domestic uses. Although OPPs are less persistent, most of them can cause harmful effects on humans [12]. Some OPPs, such as dimethoate, malathion and chlorpyrifos, have already been banned from commercialization in Europe, under Regulation EC No.1107/2009 [13].

BFRs are chemicals used to reduce the flammability of a variety of products, including electronics, vehicles, plastics and textiles. This group of chemicals consists of polybrominated diphenyl ethers (PBDEs) that comprise 209 congeners, polybrominated biphenyls (PBBs), tetrabromobisphenol A (TBBPA) and hexabromocyclododecane (HBCDD). Certain BFRs also have legislation that restricts or bans their use. Directive 2003/11/EC amends Directive 76/769/EEC and prohibits the sale of PentaBDE and OctaBDE (two commercial mixtures of PBDEs) in concentrations higher than 0.1% by mass [14]. Since 2006, all new electrical and electronic equipment should not contain PBBs and PBDEs [15,16]. In 2008, DecaBDE was also banned [17].

PCBs are a group of chlorinated hydrocarbons that include between one and ten chlorine atoms attached to a biphenyl moiety [18,19] and have 209 possible congeners. Since 1929, these chemicals have been widely used in various industrial materials such as hydraulic fluids, insulating oil in condensers and transformers and paints. Additionally, some paint manufacturing processes can produce PCB congeners as by-products [19,20]. Europe prohibited the manufacturing of PCBs in the late 1970s [19,21], the Stockholm Convention classified PCBs as persistent organic pollutants in 2001 [22] and the International Agency for the Research on Cancer (IARC) classified PCBs in Group I (carcinogenic to humans) [23].

SMs are chemicals used as fragrance additives in diverse household and personal care products, including perfumes, shampoos, lotions, deodorants, soaps and detergents. SMs can be divided into four major groups: nitro, polycyclic, macrocyclic and alicyclic musks. Nitro musks have been banned in many countries and replaced by polycyclic musks [24]. The EU forbids the use of the musks ambrette, moskene and tibetene in cosmetic products. The EU also limits the musks xylene and ketone in cosmetic products to less than 0.003–1% and 0.042–1.4%, respectively [25].

The major exposure routes for these chemicals are diet, occupational exposure, inhalation or absorption through the skin. These pollutants are linked to adverse health effects, including cancers, neurological, respiratory, immunological and reproductive disorders, thyroid dysfunction and diabetes [12,20,26,27,28,29].

These chemicals can be detected in shrimp and seawater samples all over the world [30]. Table S1 (Supplementary Material) presents some examples of contaminant levels found in Europe. The purpose of this study was to measure the presence of contaminants in two shrimp species, *Palaemon serratus* and *Palaemon varians*, and in the water from their habitat on the northwest (NW) Portuguese coast, contributing to the quality of the aquatic environment and to ensure food safety.

## 2. Results

### 2.1. Validation of the Analytical Methodology

The optimized procedure presented an adequate separation and quantification of the compounds with retention times in the range of 16.41–25.19 min (OCPs), 10.15–20.21 min (OPPs), 19.42–71.65 min (BFRs and PCBs) and 14.83–26.35 min (SMs). For all the compounds, the intraday and interday precisions were below 20%, and the results are summarized in Table S2. Intraday and interday precisions were 4.0–13.9% and 2.9–11.0% for OCPs, respectively, 6.7–9.9% and 5.4–15.8% for OPPs, respectively, 7.4–12.5% and 0.5–10.8% for BFRs, respectively, 3.3–7.7% and 1.0–4.9% for PCBs, respectively, and 7.4–13.5% and 4.1–13.2% for SMs, respectively. The results of the expanded combined uncertainty (Ur, tot) were 6.6–15.3% for OCPs, 11.3–38.0% for OPPs, 1.6–24.2% for BFRs, 5.2–15.3 for PCBs and 7.4–26.5% for SMs (Table S2). The values are in accordance with the EU guidance requirement of <50% [31].

#### 2.1.1. Water Sampled in the Shrimps’ Habitat

The optimized procedure was adequate for multiresidue analysis of various pollutants with a valuable separation and quantification of the compounds. The linearity range, coefficient of determination (R^2^), method detection (MDL) and method quantification (MQL) limits, matrix effect (ME) and average recoveries for each group (OCPs, BFRs, PCBs, OPPs and SMs) for seawater samples are presented in Table S3. Good linearity was achieved for all the curves for the entire range of concentrations with R^2^ higher than 0.99, except for BFR, TBECH (Table S3). The average recoveries presented in Table S3 ranged from 60–88% for OCPs with the exception of HCB (46%), 70–83% for OPPs, 86–93% for BFRs, 62–95% for PCBs and 65–77% for SMs. MDLs and MQLs were 0.006–0.013 ng/g wet weight (ww) and 0.020–0.044 ng/g ww for OCPs, 0.007–0.013 ng/g ww and 0.023–0.044 ng/g ww for OPPs, 0.004–0.031 ng/g ww and 0.013–0.104 ng/g ww for BFRs, 0.002–0.013 ng/g ww and 0.008–0.044 ng/g ww for PCBs and 0.009–0.013 ng/g ww and 0.029–0.045 ng/g ww for SMs, respectively. ME was calculated as described in Section 3.4, and values ranged between 16% and −109% (OCPs), 0% and 36% (OPPs), −9% and −70% (BFRs), −15% and −83% (PCBs) and 90% and 93% (SMs), as described in Table S3. For all the pollutants analyzed, 55.1% of the compounds suffered ion suppression, 18.4% ion enhancement, 24.5% were in the range of −20–20% and one presented a value of 0%.

#### 2.1.2. Shrimp Samples

The chromatographic method was optimized for the edible tissues of shrimp samples. Three different QuEChERS methods were tested for OCPs, and the best QuEChERS was the AOAC (Table S4). Applying the selected QuEChERS (AOAC), different quantities of graphitized carbon black were tested in the clean-up for OCPs, the best results were obtained with 2 mg (Table S4). The linearity range, R^2^, MDL, MQL, ME and average recoveries for each group of pollutants (OCPs, BFRs, PCBs, OPPs and SMs) are presented in Table S5. Good linearity was achieved in all analyte curves for the entire range of concentrations used, since R^2^ values were higher than 0.99. The average recoveries ranged between 56–96% for OCPs, 60–91% for OPPs, 65–136% for BFRs, 73–88% for PCBs and 74–94% for SMs (Table S5). MDLs and MQLs were 0.95–2.89 ng/g ww and 3.16–9.63 ng/g ww for OCPs, 4.23–6.39 ng/g ww and 14.10–21.31 ng/g ww for OPPs, 1.08–3.70 ng/g ww and 3.59–12.33 ng/g ww for BFRs, 1.80–3.08 ng/g ww and 5.99–10.27 ng/g ww for PCBs and 1.33–3.51 ng/g ww and 4.42–11.71 ng/g ww for SMs, respectively. The complexity of food matrices with a large number of compounds can be problematic, interfering with the analytical signal causing ME. ME values were between −9% and −212% for OCPs, 1% and 29% for OPPs, 3% and −174% for BFRs, −16% and −107% for PCBs and 38% and 61% for SMs (Table S5). For all the compounds analyzed, 49.0% of the analytes suffered ion suppression, 21.6% ion enhancement and 29.4% were in the range of −20–20%, not considered the ME [32,33].

### 2.2. Pollutants

#### 2.2.1. Pollutants in Water Sampled in the Shrimps’ Habitat

Quantification of pollutants in seawater can be challenging because the levels are potentially low in this environmental matrix. The concentrations of the analyzed pollutants found in the different locations are reported in Table 1. All the results are presented as µg/L of water sampled in the shrimps’ habitat. The OCPs found in seawater samples were α-HCH, lindane, δ-HCH, aldrin, dieldrin, DDE, DDD and DDT. However, only DDD and lindane were found above MDL. A DDD concentration of 0.012 µg/L was determined in the sample from aquaculture, in spring of 2018. Lindane was found in autumn samples from Ria de Aveiro 2017 (0.014 µg/L), Figueira da Foz 2017 (0.014 µg/L), Aveiro 2018 (0.021 µg/L) and Sado 2017 (0.023 µg/L). A previous study from 2002 also found the presence of OCPs in the Atlantic Ocean, in the Portugal zone, at 68 pg/L for α-HCH and 112 pg/L for lindane [34]. Chlorpyrifos was the only OPP found in the seawater samples analyzed, although below the MDL. A study from Zhong et al., in the North Sea reported concentrations between 27 and 86 pg/L for chlorpyrifos [35]. Regarding BFRs, BDE 28 and BTBPE were found, but only BTBPE was found above the MDL. A BTBPE concentration of 0.013 and 0.015 µg/L was found in spring samples from Ria de Aveiro 2018 and from aquaculture 2019, respectively. BFRs were detected previously in the Atlantic Ocean [36] (Table S1). BTBPE was detected previously in Korea Bays at a maximum of 0.021 ng/L [37]. PCB 28 was the only PCB found in seawater samples but was below the MDL. This is in accordance with another study in the Atlantic Ocean that found PCB 28 and other PCBs [38]. Concerning SMs, HHCB, AHTN and ketone were found in samples, although all below the MDL. A study that attempted to determine the presence of SMs in the Atlantic Ocean around Portugal did not detect any of the SMs analyzed, including HHCB, AHTN and ketone [39]. On the other hand, these three SMs were found in aquaculture sites from the Atlantic Ocean and Mediterranean Sea, with values between ˂MDL and 98.90 ng/L [40]. 

#### 2.2.2. Pollutants in Shrimp Samples

Concentrations for the analyzed pollutants found in two shrimp species in different locations are summarized in Table 2. All the results are presented as ng/g ww. Despite the fact that several OCPs were detected in water, DDD was the only OCP found in shrimp samples. A concentration of 6.09 ng/g ww of DDD was found in a sample from Vila do Conde, autumn 2017. Persistent chemicals have been reported previously in shrimp samples from Europe, and a study from Belgium reported values of not detected (ND) to 0.81 ng/g ww for HCB, DDE and HCHs [41]. Another study from Belgium–Netherlands presented values of ND to 1.03 ng/g ww for DDE, DDD and DDT [42]. A report from Italy described values of ND to 1.02 ng/g ww for DDE, DDD, DDT and HCB [43]. OPPS and BFRs were not detected in any sample analyzed. Although BFRs have been described previously [44], as reported in Table S1, for OPPs, no report from Europe on the concentration in shrimps was found (using Web of Science database). Regarding PCBs, PCB 153 and PCB 180 were detected, but below the MDL, compared to a study from the Netherlands that reported a value of 0.117 ng/g ww for the sum of PCB 28, 52, 101, 108, 138, 153 and 180 [45]. Concerning SMs, HHCB, AHTN and ketone were detected. HHCB was detected in all the samples analyzed at concentrations ranging between 3.16 ng/g ww and 7.55 ng/g ww in spring from Aveiro 2019 and Ria de Aveiro 2018, respectively. AHTN was found in three samples from spring above the MDL, 2.64 ng/g ww in Matosinhos (shrimps with eggs) 2019 and, in Ria de Aveiro and Sado from 2018, the same value was obtained, 2.97 ng/g ww. The presence of eggs or shell does not significantly affect the quantity of pollutants. Ketone was observed to be present in half of the samples, in concentrations between 2.15 ng/g ww in Ria de Aveiro and 11.06 ng/g ww in Sado from spring 2018. The concentrations found in other studies are in the range of the values reported in this work. In shrimp samples from Asia, values for HHCB and AHTN ranged from 1.5–5.3 ng/g ww [46] and a further study, analyzing shrimp samples from local supermarkets in Spain, found values for HHCB and AHTN between 2.9 and 3.8 ng/g ww [47]. The sum and average of the SMs were calculated considered MDL/$\sqrt{2}$ for concentrations below the MDL [48]. Comparing the average concentration values of spring and autumn for the three detected SMs, higher values were observed in spring. The average concentration value for HHCB was 4.89 ng/g ww in spring and 4.15 ng/g ww in autumn, and ketone presented values of 5.40 ng/g ww for spring and 4.55 ng/g ww for autumn. AHTN only presented values above the MDL in samples from spring, with an average of 2.86 ng/g ww. Evaluating the difference between the values for spring and autumn for the three SMs, we found that HHCB was significantly higher in spring samples (*p* = 0.027). Regarding the two shrimp species, the average for *P. serratus* was 4.64, 1.86 and 4.41 ng/g ww and for *P. varians* it was 4.40, 1.83 and 6.33 ng/g ww for HHCB, AHTN and ketone, respectively. Aquaculture shrimps have slightly lower values of HHCB and partially higher values of AHTN and ketone.

### 2.3. Bioaccumulation

The bioaccumulation degree can be reported as the bioaccumulation factor (BAF). For the calculation of the BAF, only the values detected for SMs were used, because these were found simultaneously in water and shrimp samples between 2017 and 2019. All SMs detected, HHCB, AHTN and ketone in seawater samples were below the MDL, whereas in shrimp samples HHCB and ketone were all above the MDL and three samples also presented AHTN above the MDL. For the pollutants below the MDL, the MDL was used to calculate the BAF [49]. The BAF values calculated are presented in Table 3. We observed bioaccumulation of all SMs detected in the biota samples. Regarding the average values for the three SMs detected, the BAF values are ranked as follows: ketone > HHCB > AHTN, ranging between 183.2 L/kg ww for *P. serratus* and 940.7 L/kg ww for *P. varians*. The BAF values regarding SMs in fish samples reported previously showed a wide range, from 93.3 L/kg ww to 6,456.5 L/kg ww, suggesting species-specific accumulation patterns [50,51].

### 2.4. Maximum Admissible Concentration

Pollutants such as OCPs, OPPs, PCBs and BFRs are already banned or have been restricted (with maximum levels legislated) [2,52,53,54]. The concentrations detected in this study for these pollutants were almost all ˂ MDL, with the exception of lindane, DDD and BTBPE in seawater samples and DDD in shrimp samples. We can see that OCPs such as DDD and lindane, already banned for several years, can still be detected in the samples analyzed with values > MDL. BTBPE, detected in seawater samples, is an emerging BFR and, although it has been investigated, it is not yet legislated. The limited experimental data collected for BTBPE identified that this compound can accumulate in the body over time, due to the BAFs reported. These findings were included in a scientific opinion released by the European Food Safety Authority (EFSA) [55]. Regarding SMs, HHCB, ANTH and ketone were detected in shrimp samples > MDL. Although some legislation has been established, concerning the maximum concentration allowed of SMs in cosmetics [25], no regulation has been established for environmental or biota samples. The results of this study reported concentrations of SMs in shrimps and with BAF values higher than 100. These findings validate that specific legislation for SMs should be enforced for foodstuffs, such as fishery products.

### 2.5. Risk Assessment

The risk of exposure was evaluated for the detected SMs (HHCB, AHTN and ketone), the group of contaminants present with higher frequency in the analyzed samples. Data on safe SM consumption levels and their health risks for human population are very limited. However, to these SMs are attributed values for no observed adverse effect level (NOAEL) [56,57,58], as presented in Table 4. Based on this, the tolerable daily intake (TDI) was calculated. The values for maximum shrimp daily consumption per individual (28.82 g/day) and mean adult body weight in the Portuguese population (71.68 kg) were described in a previous work reporting data on the Portuguese population [59]. Only values of SM concentrations above the MDL were used to calculate the dietary exposure. The results described in Table 4 present values of exposure (median, 25th and 75th percentile) all below the TDI calculated, so it is unlikely that a potential health risk is present based on this risk assessment. These results are in accordance with previous works reporting risk assessment for SMs in seafood [46,47].

## 3. Materials and Methods

In this study, fourteen OCPs, six OPPs, twelve BFRs, thirteen PCBs and six SMs were included, in a total of 51 endocrine disruptors. Most of these compounds have high Log Kow which increases the potential to bioconcentrate in living organisms.

### 3.1. Sample Collection

*P. serratus* and *P. varians* shrimp species were collected in Portugal. *P. serratus* was collected along the NW Portuguese coast (namely in Vila do Conde, Matosinhos, Aveiro, Ria de Aveiro and Figueira da Foz) by local fishermen and *P. varians* (wild and aquaculture origin) was collected in the Sado estuary. *P. serratus*’s size ranged from 4–9 cm and the size of *P. varians* ranged from 2–4 cm. A total of 1 kg of shrimps was sampled in each location. The sampling was performed in autumn and spring between 2017 and 2019 in all the locations, according to the period allowed for shrimp capture by Portuguese law [60]. The edible portion of the shrimp was separated from the shell, except in some samples of *P. varians* in which separation was not possible, due to the small size of the shrimp; shrimps with eggs were stored separately. Twenty-eight samples were storage at −20 °C until analysis.

Surface water was collected in the same locations of shrimp samples and during the same period. A total of twenty-five samples were gathered: 4 in Vila do Conde, 4 in Matosinhos, 3 in Aveiro, 2 in Ria de Aveiro, 4 in Figueira da Foz and 8 in the Sado estuary (4 for aquaculture and 4 for wild shrimp). Samples were collected in clean glass bottles, previously rinsed with ultra-pure water and stored at −20 °C until extraction.

### 3.2. Reagents, Solvents and Materials

The detailed description of the reagents, solvents and materials used for the extraction and analysis of the studied contaminants is given in Table S6. For the contaminants analyzed by GC (Table S7), standard stock solutions of individual and mixed compounds were prepared in n-hexane with different concentrations and stored in amber colored vials at 4 °C prior to utilization.

### 3.3. Extraction Procedure

#### 3.3.1. Water Sampled in the Shrimps’ Habitat

Prior to extraction, the samples were filtered with ashless filter paper and the sample pH was adjusted to 2.5 using HCl 1 M. Internal standard (IS) was added to volumetric flasks (final concentrations of 100 µg/L for 4.4’-dichlorobenzophenone and 50 µg/L for 5′-fluoro-2,3′,4,4′,5-pentabromodiphenyl ether, TPP and AHTN-d3). The solid phase extraction (SPE) method was used for the extraction procedure. Strata C18-E cartridges (500 mg, 3 mL), in a vacuum system manifold (Chromabond, Düren, Germany), were previously cleaned with 5 mL of ethyl acetate:dichloromethane (1:1), and conditioned with 5 mL of methanol and 5 mL of ultrapure water. Subsequently, seawater samples were percolated through the cartridge. Then, the cartridges were rinsed with 10 mL of ultrapure water and dried under vacuum for 15 min. The elution was performed using 8 mL of ethyl acetate and 8 mL of dichloromethane. The cartridges were dried under vacuum. Anhydrous sodium sulfate (5 g) was added to the eluted samples (to remove all the existent water). In the end, 30 µL of *D*-(+)-gluconic acid δ-lactone at 5 mg/mL in acetonitrile (ACN)/ultrapure water (9:1) were added as an analyte protector. The extract was evaporated under a stream of nitrogen and the residues were reconstituted with 500 µL of *n*-hexane and injected into the chromatograph. OCPs, BFRs and PCBs were analyzed using gas chromatography with an electron capture detector (GC-ECD), OPPs by gas chromatography with a flame photometric detector (GC-FPD) and SMs using gas chromatography coupled with a mass spectrometric detector (GC-MS). Positive results were confirmed by GC-MS/MS. Details of the chromatography analysis are presented in the Supplementary Material (Section S1), GC-MS and MS/MS conditions for SMs are described in Table S8 [61,62] and GC-MS/MS conditions for the other compounds in Table S9 [63,64,65].

#### 3.3.2. Shrimp

Optimization of the extraction procedure was performed with OCPs, by testing different QuEChERS (Original, AOAC and EN) and clean-up compositions (150 mg anhydrous magnesium sulfate, 50 mg PSA and 50 mg C18 with 2 mg graphitized carbon black or 5 mg graphitized carbon black) (Table S4). The optimized method was then tested and applied to all analyzed contaminants. Briefly, shrimp edible portions (shrimp muscle) were mechanically homogenized with a kitchen blender (Braun, Frankfurt, Germany), 5 g were weighed and the IS solution was added to a final concentration of 100 µg/L for 4.4′-dichlorobenzophenone and 50 µg/L for 5′-fluoro-2,3′,4,4′,5-pentabromodiphenyl ether, TPP and AHTN-d3. Next, 8 mL of ACN were added to the shrimp, followed by vortex homogenization for 3 min. QuEChERS AOAC was added and the mixture was again vortexed for 3 min and then centrifuged at 4000 rpm for 5 min. Afterwards, 1 mL of supernatant was transferred to a 2 mL tube containing the clean-up sorbents (150 mg anhydrous magnesium sulfate, 50 mg PSA, 50 mg C18 and 2 mg graphitized carbon black). The tubes were vortexed for 2 min followed by centrifugation at 4000 rpm for 5 min. An aliquot of the supernatant 300 µL (for OCP and OPPs) or 600 µL (for BFRs, PCBs and SMs) was transferred to a vial and concentrated just to dryness using a gentle stream of nitrogen. The sample residue was reconstituted in 150 µL of n-hexane and injected into the GC for analysis. GC analysis of shrimp samples was conducted as described for seawater samples.

### 3.4. Method Validation

Repeatability was presented as intraday precision and intermediate as interday precision. Calibration curves were obtained for all contaminants analyzed in this study in shrimp or seawater fortified after extraction with standard solutions (matrix-matched calibration). In seawater samples, recoveries were calculated using spiked samples at three levels in triplicate, 0.06, 0.09 and 0.13 µg/L for all the compounds analyzed. The recoveries for shrimp samples were calculated using spiked samples at three levels, 16, 24 and 32 ng/g *w*/*w* for OCPs, 16, 28 and 40 ng/g *w*/*w* for OPPs and 8, 14 and 20 ng/g *w*/*w* for BFRs, PCBs and SMs, with each level in triplicate. The ME was calculated, as previously described [32], using standard solutions prepared in n-hexane compared to spiked blank samples. A positive result corresponds to an enhancement of the analytical response, whereas a negative value corresponds to a suppression effect. Values within the 20% signal variation are not considered as a ME [32,33]. The uncertainty was determined following the “top-down” approach, using validation data and the uncertainty of the purity of analytical standards. The coverage factor k of 2 and confidence level of 95% were applied for all the analytes [32].

### 3.5. Bioaccumulation Factor 

The BAF can be measured as the ratio between concentration in aquatic species (shrimp μg/kg ww) and the concentration in ambient water (µg/L) [49]. The units of the BAF are mostly presented as L/kg [66]. A BAF greater than 1 indicates a potential of the organism to accumulate the pollutants, and values above 100 are considered significant [67].

### 3.6. Risk Assessment

A risk assessment was performed for a group of contaminants present in a higher frequency in the analyzed samples. The NOAEL [56,57,58] values were used to calculate a TDI by applying an uncertainty factor of 100 to account for the human variability and species differences [46]. The dietary exposure (µg/kg bw/day) of SMs was calculated according to the Equation (1) described previously [47].

(1) $$Ei=\frac{Ci}{B}\times x$$

where *Ei* is the dietary exposure to the SM for individual (*i*) (µg/kg bw/day), *Ci* is the maximum shrimp daily consumption by individual (*i*) (g/day), *B* is the mean body weight of the adult Portuguese population (kg) and *x* is the concentration of SM in shrimp samples (μg/kg).

### 3.7. Statistical Analysis

The statistical analysis was performed using GraphPad Prism 6.01 software (La Jolla, CA, USA). Differences were considered significant when *p* < 0.05. Statistical significance of the difference between groups (autumn and spring) was evaluated by Student’s *t*-test and a Wilcoxon test.

## 4. Conclusions

This study reports, for the first time, the analysis of trace organic pollutants in shrimp species collected from the NW Portuguese coast. Lindane, DDD and BTBPE were detected above the MDL in seawater samples. In shrimp samples, DDD, HHCB, AHTN and ketone were found above the MDL. The average concentrations of SMs in shrimp samples were higher in spring than in autumn. In addition, aquaculture shrimps had lower HHCB values and higher AHTN and ketone values than wild shrimp. Bioaccumulation of HHCB, AHTN and ketone was confirmed using BAF calculation. The estimated exposure levels of SMs in shrimp samples were all below the estimated TDI, although these results need to be interpreted with caution due to the uncertainties of the limited data available. Although some of these pollutants have been banned for several years, they can still be detected in the environment today. Our results highlight the importance of continuous monitoring of pollutants in aquatic environments and marine organisms. For emerging pollutants like SMs, it is essential to establish specific legislation for aquatic environments and fishery products, such as shrimp, which is highly valuable in the human diet.

## Figures and Tables

**Table 1:** Levels for the pollutants found in water sampled in the shrimps’ habitat (µg/L) from NW Portuguese coast; ND: not detected.

	Concentrations µg/L														
	OCPs								OPPs	BFRs		PCBs	SMs		
Location and Date of Sampling	α-HCH	Lindane	δ-HCH	Aldrin	DDE	Dieldrin	DDD	DDT	Chlorpyrifos	BDE 28	BTBPE	PCB 28	HHCB	AHTN	Ketone
Vila do Conde Autumn 2017	ND	ND	ND	˂MDL	ND	ND	ND	ND	˂MDL	ND	ND	ND	˂MDL	˂MDL	˂MDL
Vila do Conde Spring 2018	˂MDL	˂MDL	˂MDL	ND	ND	ND	ND	ND	ND	ND	ND	˂MDL	˂MDL	˂MDL	˂MDL
Vila do Conde Autumn 2018	˂MDL	˂MDL	˂MDL	ND	ND	ND	ND	ND	ND	ND	ND	ND	˂MDL	˂MDL	˂MDL
Vila do Conde Spring 2019	˂MDL	ND	ND	ND	ND	ND	ND	ND	ND	ND	ND	ND	˂MDL	˂MDL	ND
Matosinhos Autumn 2017	ND	ND	ND	ND	ND	ND	˂MDL	˂MDL	ND	ND	ND	ND	˂MDL	˂MDL	˂MDL
Matosinhos Spring 2018	ND	˂ MDL	ND	ND	ND	ND	ND	ND	ND	ND	ND	ND	˂MDL	˂MDL	˂MDL
Matosinhos Autumn 2018	˂MDL	ND	ND	ND	ND	ND	ND	ND	ND	ND	ND	ND	˂MDL	˂MDL	˂MDL
Matosinhos Spring 2019	ND	˂MDL	ND	ND	ND	ND	ND	ND	ND	ND	ND	ND	˂MDL	˂MDL	˂MDL
Aveiro Spring 2018	ND	˂MDL	ND	˂MDL	ND	ND	ND	ND	ND	ND	ND	ND	˂MDL	˂MDL	˂MDL
Aveiro Autumn 2018	ND	0.021	ND	ND	ND	ND	ND	ND	ND	ND	ND	ND	˂MDL	˂MDL	˂MDL
Aveiro Spring 2019	ND	˂MDL	˂MDL	ND	ND	˂MDL	ND	ND	ND	ND	ND	ND	˂MDL	˂MDL	˂MDL
Ria de Aveiro Autumn 2017	ND	0.014	ND	˂MDL	ND	ND	ND	ND	ND	ND	ND	ND	˂MDL	˂MDL	˂MDL
Ria de Aveiro Spring 2018	ND	ND	ND	ND	ND	ND	ND	ND	ND	ND	0.013	ND	˂MDL	˂MDL	˂MDL
Figueira da Foz Autumn 2017	ND	0.014	˂MDL	ND	ND	ND	ND	ND	ND	ND	ND	ND	˂MDL	˂MDL	˂MDL
Figueira da Foz Spring 2018	ND	ND	˂MDL	ND	ND	ND	ND	ND	ND	ND	ND	ND	˂MDL	˂MDL	˂MDL
Figueira da Foz Autumn 2018	ND	˂MDL	˂MDL	ND	ND	ND	ND	ND	ND	ND	ND	ND	˂MDL	˂MDL	˂MDL
Figueira da Foz Spring 2019	ND	ND	ND	˂MDL	˂MDL	ND	ND	ND	ND	˂MDL	ND	ND	˂MDL	˂MDL	˂MDL
Aquaculture Autumn 2017	ND	ND	ND	ND	ND	ND	ND	ND	˂MDL	ND	ND	ND	˂MDL	˂MDL	ND
Aquaculture Spring 2018	ND	ND	˂MDL	ND	ND	ND	0.012	ND	ND	ND	ND	ND	˂MDL	˂MDL	˂MDL
Aquaculture Autumn 2018	ND	ND	ND	ND	ND	ND	ND	ND	ND	ND	ND	ND	˂MDL	ND	˂MDL
Aquaculture Spring 2019	ND	ND	ND	ND	ND	ND	ND	ND	ND	ND	0.015	ND	˂MDL	˂MDL	˂MDL
Sado Autumn 2017	˂MDL	0.023	˂MDL	˂MDL	ND	ND	ND	ND	ND	ND	ND	ND	˂MDL	˂MDL	˂MDL
Sado Spring 2018	ND	ND	ND	ND	ND	ND	ND	ND	ND	ND	ND	ND	˂MDL	˂MDL	˂MDL
Sado Autumn 2018	ND	ND	ND	ND	ND	ND	ND	ND	ND	ND	ND	ND	˂MDL	˂MDL	˂MDL
Sado Spring 2019	ND	ND	ND	ND	ND	ND	ND	ND	ND	ND	ND	ND	˂MDL	˂MDL	˂MDL

**Table 2:** Levels for the pollutants found in shrimp samples (ng/g ww) from Portugal.

	Concentrations ng/g ww					
	OCPs	PCBs		SMs		
Location and Date of Sampling	DDD	PCB 153	PCB 180	HHCB	AHTN	Ketone
Vila do conde Autumn 2017	6.09	ND	ND	3.34	ND	3.59
Vila do Conde Spring 2018	ND	ND	ND	4.76	ND	ND
Vila do Conde Spring 2018_E	ND	ND	˂MDL	5.26	ND	5.38
Vila do Conde Autumn 2018	ND	ND	ND	4.17	ND	ND
Vila do Conde Spring 2019	ND	ND	ND	4.34	˂MDL	ND
Vila do Conde Spring 2019_E	ND	ND	ND	3.95	ND	ND
Matosinhos Autumn 2017	ND	˂MDL	ND	3.49	˂MDL	ND
Matosinhos Spring 2018_E	ND	ND	˂MDL	4.95	ND	6.71
Matosinhos Autumn 2018	ND	ND	ND	5.78	ND	ND
Matosinhos Spring 2019	ND	ND	ND	5.92	˂MDL	ND
Matosinhos Spring 2019_E	ND	ND	ND	4.30	2.64	ND
Aveiro Spring 2018	ND	ND	˂MDL	5.38	˂MDL	5.49
Aveiro Autumn 2018	ND	ND	ND	3.64	ND	ND
Aveiro Spring 2019	ND	ND	ND	3.16	˂MDL	4.62
Ria de Aveiro Atumn 2017	ND	ND	ND	4.74	ND	ND
Ria de Aveiro Spring 2018	ND	ND	ND	7.55	2.97	2.15
Figueira da Foz Autumn 2017	ND	ND	ND	3.76	˂MDL	4.52
Figueira da Foz Spring 2018	ND	ND	ND	4.75	ND	ND
Figueira da Foz Autum 2018	ND	ND	ND	5.30	ND	3.30
Figueira da Foz Spring 2019	ND	ND	ND	4.18	ND	3.96
Aquaculture Autumn 2017	ND	ND	ND	4.02	ND	7.41
Aquaculture Spring 2018	ND	ND	ND	6.02	ND	4.26
Aquaculture Autumn 2018	ND	ND	ND	4.46	˂MDL	3.96
Aquaculture Spring 2019_S	ND	ND	ND	3.79	˂MDL	4.95
Sado Autumn 2017	ND	ND	ND	3.17	˂MDL	ND
Sado Spring 2018	ND	ND	ND	6.34	2.97	11.06
Sado Autumn 2018_S	ND	ND	˂MDL	3.85	ND	ND
Sado Spring 2019_S	ND	˂MDL	˂MDL	3.58	˂MDL	ND

ND: not detected; E: eggs; S: shell.

**Table 3:** Bioaccumulation factors (BAFs) for the SMs found in shrimps (L/kg ww).

Location and Date of Sampling	HHCB	AHTN	Ketone
Vila do conde Autumn 2017	249.4		305.2
Vila do Conde Spring 2018	355.7		
Vila do Conde Autumn 2018	312.0		
Vila do Conde Spring 2019	324.1	254.2	
Matosinhos Autumn 2017	260.9	254.2	
Matosinhos Spring 2018	369.9		570.6
Matosinhos Autumn 2018	432.4		
Matosinhos Spring 2019	442.8	254.2	
Aveiro Spring 2018	402.3	254.2	467.3
Aveiro Autumn 2018	272.5		
Aveiro Spring 2019	236.0	254.2	392.6
Ria de Aveiro Autumn 2017	354.6		
Ria de Aveiro Spring 2018	564.5	346.3	183.2
Figueira da Foz Autumn 2017	281.4	254.2	384.7
Figueira da Foz Spring 2018	354.9		
Figueira da Foz Autumn 2018	396.4		280.3
Figueira da Foz Spring 2019	312.7		336.7
Aquaculture Autumn 2017	300.6		
Aquaculture Spring 2018	450.3		362.1
Aquaculture Autumn 2018	333.7		336.4
Aquaculture Spring 2019	283.4	254.2	421.3
Sado Autumn 2017	237.1	254.2	
Sado Spring 2018	474.1	345.6	940.7
Sado Autumn 2018	288.0		
Sado Spring 2019	267.7	254.2	

**Table 4:** NOAEL values and the estimated exposure to detected SMs from shrimp consumption among the Portuguese population.

	NOAEL	TDI Calculated	Median Exposure	P25 Exposure	P75 Exposure
SMs	mg/kg bw/day	µg/kg bw/day	µg/kg bw/day	µg/kg bw/day	µg/kg bw/day
HHCB	50	500	1.74	1.52	2.13
AHTN	5	50	1.19	1.06	1.20
Ketone	2.5	25	1.84	1.55	2.33

## Data Availability

Not applicable.

## Supplementary Materials

The following are available online. Section S1: Gas chromatography Analysis description; Table S1: Concentration levels of the contaminants found in shrimp (ng/g ww) and seawater (μg/L) samples in previous studies in Europe; Table S2: Summary of uncertainties, intraday and interday precision obtained for OCPs, OPPs, BFRs, PCBs and SMs analytes; Table S3: Validation parameters using water sampled in the shrimp’s habitat matrix matched calibration for the selected OCPs, OPPs, BFRs, PCBs and SMs; Table S4: QuEChERS and clean-up optimization; Table S5: Validation parameters using shrimp matrix matched calibration for the selected OCPs, OPPs, BFRs, PCBs and SMs; Table S6: Reagents, solvents, and materials used in the extraction of the samples and in the analysis of the studied contaminants; Table S7: Physic-chemical characteristics, molecular mass, and the supplier company of the studied contaminants; Table S8: GC-MS and MS/MS conditions for SMs analysis; Table S9: GC-MS/MS conditions for OCPs, OPPs, BFRs, PCBs analysis.

## Sample Availability

Samples of the compounds are not available from the authors.
