# Supplementary material for: Validation and Evaluation of Selected Organic Pollutants in Shrimp and Seawater Samples from the NW Portuguese Coast
Source: Molecules. 2021 Sep 23;26(19):5774. doi: 10.3390/molecules26195774 (PMC8510022; doi:10.3390/molecules26195774)
Supplement: Supplementary file 1 [file molecules-26-05774-s001.zip › molecules-1391108-supplementary.pdf]

Supplementary material

Validation and evaluation of selected organic pollutants in shrimp and seawater samples from the NW Portuguese coast

Maria Luz Maia[1,2], Cristina Delerue-Matos[1], Conceição Calhau[2,3], Valentina Fernandes Domingues[1]

[1] REQUIMTE/LAQV-GRAQ, Instituto Superior de Engenharia do Porto, Politécnico do Porto, 4249-015 Porto, Portugal

[2] Center for Research in Health Technologies and Information Systems, 4200-450 Porto, Portugal

[3] Nutrição e Metabolismo, NOVA Medical School, Faculdade de Ciências Médicas Universidade Nova de Lisboa, 1169-056 Lisboa, Portugal

Section 1 - Gas chromatography Analysis description – P2

Table S1 - Concentration levels of the contaminants found in shrimp (ng/g ww) and seawater (µg/L) samples in previous studies in Europe – P3

Table S2 - Summary of uncertainties, intraday and interday precision obtained for OCPs, OPPs, BFRs, PCBs and SMs analytes – P4

Table S3 - Validation parameters using water sampled in the shrimp's habitat matrix matched calibration for the selected OCPs, OPPs, BFRs, PCBs and SMs – P5

Table S4 - QuEChERS and clean-up optimization – P6

Table S5 - Validation parameters using shrimp matrix matched calibration for the selected OCPs, OPPs, BFRs, PCBs and SMs – P7

Table S6 - Reagents, solvents, and materials used in the extraction of the samples and in the analysis of the studied contaminants – P8

Table S7 - Physic-chemical characteristics, molecular mass, and the supplier company of the studied contaminants – P9

Table S8 - GC-MS and MS/MS conditions for SMs analysis – P11

Table S9 - GC-MS/MS conditions for OCPs, OPPs, BFRs, PCBs analysis – P11

Section 1

Gas chromatography Analysis description

Gas Chromatography – Electron Capture Detector

OCPs, BFRs and PCBs analysis were carried out with a Shimadzu GC-2010 (Kyoto, Japan) with an ECD detector, equipped with capillary Zebron-XLB column of 30 m (0.25 mm i.d., 0.25 µm film thickness, Phenomenex, California, USA). The carrying gas, helium, from Linde Sogás (purity ≥99.999%), flowed at 1.66 mL/min with a linear velocity of 36 cm/s. Whereas nitrogen (Linde Sogás, purity ≥99.999%) was employed as makeup gas at a flow rate of 30 mL/min. Injection was carried out in splitless mode, with an injector temperature of 250 °C and detector temperature was set at 300 ºC. The system was operated by GC-Solutions Shimadzu software. For OCPs determination, the column temperature programmed was the follow: start at 40 ºC (1 min hold), followed by increases of 20 ºC/min to 120 ºC (1 min hold), 10 ºC/min to 150 ºC (1 min hold), 10 ºC/min to 180 ºC (1 min hold), 20 ºC/min to 200 ºC (1 min hold) and 10 ºC/min to 290 ºC (2 min hold). For BFRs and PCBs analysis, the temperature program start at 40 ºC (1 min hold) followed by increases of 15 ºC/min to 120 ºC (1min hold), 10 ºC/min to 150 ºC (1 min hold), 10 ºC/min to 180 ºC (1 min hold), 20 ºC/min to 200 ºC (1 min hold), 2 ºC/min to 210 ºC (1 min hold), 2 ºC/min to 220 (2 min hold), 2 ºC/min to 260 ºC (2 min hold), 0.5 ºC/min to 270 ºC (2 min hold), 2ºC/min to 280 ºC.

Gas Chromatography - Flame Photometric Detector

OPPs analysis was carried out with a Shimadzu GC-2010 (Kyoto, Japan) with an FPD detector with phosphorus filter equipped with a TRB-5 column of 25 m (0.25 mm i.d., 0.18 µm film thickness, Teknokroma, Barcelona, Spain). The carrying gas, helium, from Linde Sogás (purity ≥99.999%), flowed at 0.89 mL/min with a linear velocity of 25.4 cm/s. The injector temperature was 250 °C and operated in splitless mode and the detector was maintained at 290 ºC. The system was operated by GC Solution Shimadzu software. The GC oven temperature was programmed for an initial temperature of 100 ºC (1 min hold), increasing at 20 ºC/min to 150 ºC (1 min hold), 2 ºC/min to 180 ºC (2 min hold) and finally at 20 ºC/min to 270 ºC (3 min hold).

Gas Chromatography - Tandem Mass Spectrometry

SMs analysis were performed using a Trace GC Ultra gas chromatograph Polaris Q coupled with ion trap mass spectrometer (Thermo Fisher Scientific, Massachusetts, USA) operated in the electron impact ionization mode (70eV), equipped with Zebron ZB-5MSi column 30 m (0.25 mm i.d., 0.25 µm film thickness, Phenomenex,

California, USA). Operated by Xcalibur 1.3 software. Quantification was performed in selected ion monitoring (SIM) mode. The samples were injected through a splitless mode with helium from (Linde Sógas, purity ≥99.999%) as carrier gas at a constant flow rate of 1.3 mL/min. The injector was maintained at 240ºC. The GC oven temperature was programmed from an initial temperature of 80ºC, increasing at 10ºC/min to 160 ºC (10 min hold) and finally at 3 ºC/min to 200 ºC (1 min hold).

Table S1 - Concentration levels of the contaminants found in shrimp (ng/g ww) and seawater (µg/L) samples in previous studies in Europe.

			Σ concentrations (number of contaminants detected)					
	**Location**	**Date**	**OCPs**	**OPPs**	**BFRs**	**PCBs**	**SMs**	**Ref**
Shrimp	Scheldt Estuary (Belgian)	2001	ND - 2.61 (5)					[41]
Shrimp	Tyrrhenian Sea in the Gulf of Naples	2003 - 2004	ND - 1.43 (4)					[43]
Shrimp	Suppliers from Netherlands	2007 - 2008				0.117 (7)		[45]
Shrimp	Scheldt estuary (Netherlands–Belgium)	2010	ND - 1.37 (3)		0.48 (3)			[42]
Shrimp	Scheldt Estuary (Netherlands)	2001			35.05 – 47.49 (17)			[44]
Shrimp	Establishments in Tarragona, Spain						11.5 (2)	[47]
	Atlantic Ocean, Portuguese Coast	2017 - 2018	ND - 6.09 (1)	ND	ND	ND	3.49 - 12.67 (3)	This study
	Atlantic Ocean, Aquaculture Portugal	2017 - 2018	ND	ND	ND	ND	3.17 - 20.37 (3)	This study
Seawater	Atlantic Ocean, Portugal	1997	0.000180 (2)					[34]
Seawater	Atlantic Ocean	2005				0.0000037 - 0.000220 (8)		[38]
Seawater	Atlantic Ocean	2009			ND - 0.0000072 (10)			[36]
Seawater	North Sea, Atlantic Ocean	2010	0.000065 (5)	0.000061 (1)				[35]
Seawater	Atlantic Ocean and Mediterranean Sea	2016					ND - 0.306 (4)	[40]
Seawater	Atlantic Ocean, Portugal	2017					ND (5)	[39]
	Atlantic Ocean, Portuguese Coast	2017 - 2018	ND - 0.021 (1)	ND	ND - 0.013 (1)	ND	ND	This study
	Atlantic Ocean, Aquaculture Portugal	2017 - 2018	ND - 0.023 (2)	ND	ND - 0.015 (1)	ND	ND	This study

Table S2 - Summary of uncertainties, intraday and interday precision obtained for OCPs, OPPs, BFRs, PCBs and SMs analytes.

Analyte	ucm	ur,repro (%)	Br (%)	ur,cm (%)	ur,ref (%)	u r,tot (%)	Ur,tot (%)	Intraday (%)	Interday (%)
α-HCH	0.7	3.4	-5.26	1.4	0.3	6.4	12.9	11.4	4.0
HCB	0.6	3.0	-0.66	1.2	0.3	3.3	6.6	9.7	2.9
β-HCH	1.0	3.8	3.07	2.0	0.3	5.3	10.5	4.9	10.1
Lindane	1.0	4.2	-1.56	2.1	0.3	4.9	9.9	13.9	7.6
δ-HCH	0.5	1.8	-3.65	1.0	0.3	4.2	8.3	12.7	11.0
Aldrin	0.6	3.1	1.07	1.3	0.3	3.5	7.0	5.3	2.9
α-endosulfan	1.4	6.1	-0.83	2.7	0.3	6.7	13.4	4.0	6.0
DDE	0.7	3.2	-0.06	1.4	0.3	3.5	7.0	8.7	4.4
Dieldrin	0.8	3.3	1.14	1.5	0.3	3.8	7.7	6.7	4.6
Endrin	0.8	3.0	1.59	1.5	0.3	3.7	7.4	4.0	6.6
DDT	1.3	6.1	-3.69	2.6	0.3	7.6	15.3	5.4	11.3
DDD	1.1	4.2	-0.68	2.1	0.3	4.8	9.6	4.2	5.2
β-endosulfan	1.4	5.7	0.24	2.9	0.3	6.4	12.8	6.7	6.0
Methoxychlor	1.0	4.8	-2.78	2.1	0.3	5.9	11.8	4.9	7.4
Dimethoate	1.6	7.6	-5.01	3.2	0.3	9.7	19.4	9.9	8.7
Chlorpyrifos-methyl	1.3	6.0	-0.93	2.7	0.3	6.7	13.3	7.4	7.9
Parathion-methyl	0.9	4.2	-3.21	1.8	0.3	5.6	11.3	9.4	5.4
Malathion	2.0	9.3	-1.77	4.1	0.3	10.3	20.6	8.2	9.5
Chlorpyrifos	1.9	8.3	-0.04	3.7	0.3	9.1	18.2	6.7	8.1
Chlorfenvinphos	3.5	16.5	-6.37	6.9	0.3	19.0	38.0	8.1	15.8
TBECH	1.3	5.3	5.05	2.5	0.3	7.7	15.5	7.4	6.6
BDE 28	1.6	7.2	9.22	3.2	0.3	12.1	24.2	10.6	5.9
PBT	0.5	2.5	2.85	1.1	0.3	4.0	7.9	12.4	2.2
PBEB	0.9	4.0	3.17	1.7	0.3	5.4	10.8	11.4	3.4
BDE 47	0.7	2.9	4.66	1.3	0.3	5.6	11.3	9.9	2.7
BDE 100	0.7	3.1	2.83	1.3	0.3	4.4	8.9	10.4	2.6
BDE 99	0.1	0.6	0.38	0.3	0.3	0.8	1.6	8.0	0.5
TBB	0.7	3.1	3.37	1.3	0.3	4.8	9.6	8.5	2.6
BDE 153	0.7	3.2	-1.73	1.3	0.3	3.9	7.8	12.3	2.8
BDE 154	1.2	5.7	-2.37	2.5	0.3	6.6	13.2	12.5	5.8
BDE 183	1.4	5.7	-3.29	2.8	0.3	7.2	14.3	11.3	6.8
BTBTE	1.0	4.5	-0.08	2.0	0.3	5.0	10.0	10.7	10.8
PCB 28	0.4	1.6	4.97	0.7	0.3	5.3	10.5	3.8	2.4
PCB 52	0.5	2.4	4.84	1.0	0.3	5.5	11.0	4.6	1.9
PCB 101	0.9	3.7	6.40	1.8	0.3	7.6	15.3	7.7	4.9
PCB 77	0.6	2.6	5.56	1.1	0.3	6.2	12.5	5.3	2.1
PCB 118	0.8	3.5	-0.73	1.6	0.3	3.9	7.8	3.3	3.4
PCB 114	0.8	3.5	5.71	1.5	0.3	6.8	13.7	6.1	3.0
PCB 153	0.4	1.8	5.96	0.8	0.3	6.3	12.6	7.0	1.5

PCB 138	0.6	2.7	6.25	1.2	0.3	6.9	13.8	3.9	2.1
PCB 126	0.4	1.7	3.71	0.8	0.3	4.2	8.4	7.2	1.6
PCB 156	0.3	1.6	1.86	0.7	0.3	2.6	5.2	6.6	1.4
PCB 157	0.6	2.7	3.29	1.1	0.3	4.4	8.8	4.8	2.1
PCB 180	0.2	1.2	2.92	0.5	0.3	3.2	6.4	6.2	1.0
PCB 169	0.6	2.5	2.79	1.2	0.3	4.0	7.9	5.0	2.3
celestolide	1.3	6.6	-0.99	2.7	0.3	7.2	14.4	11.3	8.3
ambrette	0.8	3.4	-7.12	1.6	0.3	8.1	16.1	10.8	13.0
HHCB	1.7	7.5	-0.73	3.3	0.3	8.2	16.4	11.5	13.2
xylene	1.3	6.7	-11.11	2.7	0.3	13.2	26.5	11.6	7.3
AHTN	0.6	2.4	2.58	1.1	0.3	3.7	7.4	13.5	5.0
ketone	0.6	3.1	-6.78	1.3	0.3	7.6	15.1	7.4	4.1

*u*cm- standard uncertainty of mean measured; *u*r,repro – within-lab reproducibility; *B*r - relative bias; *u*r,cm – uncertainty of systematic error; *u*r,ref – uncertainty of purity of analytical standard; *u*r,tot - relative combined measurement uncertainty; *U*r,tot - expanded combined measurement uncertainty.

Table S3 - Validation parameters using water sampled in the shrimp's habitat matrix matched calibration for the selected OCPs, OPPs, BFRs, PCBs and SMs.

		Calibration range (µg/L)	Regression equation (µg/L)	Linearity (R^2)	MDL (µg/L)	MQL (µg/L)	ME	Recoveries
OCPs	α-HCH	0.01 - 0.13	y= 132893779x + 1021457	0.997	0.008	0.026	-88%	60%
	HCB	0.01 - 0.13	y= 49374711x + 796376	0.993	0.013	0.044	-102%	46%
	β-HCH	0.01 - 0.13	y= 12377955x + 189596	0.994	0.011	0.036	-76%	87%
	Lindane	0.01 - 0.13	y= 113396890x + 1965367	0.995	0.009	0.031	-63%	72%
	δ-HCH	0.01 - 0.13	y= 104216354x + 1075640	0.995	0.010	0.033	-109%	84%
	Aldrin	0.01 - 0.13	y= 65240137x + 301274	0.998	0.006	0.021	-74%	75%
	α-endosulfan	0.01 - 0.13	y= 47758982x + 284167	0.993	0.011	0.037	-52%	81%
	DDE	0.01 - 0.13	y= 39964569x + 19073440	0.991	0.013	0.042	-24%	80%
	Dieldrin	0.01 - 0.13	y= 60109710x + 401794	0.994	0.011	0.036	-32%	79%
	Endrin	0.01 - 0.13	y= 42515476x + 196073	0.992	0.012	0.040	16%	81%
	β-endosulfan	0.01 - 0.13	y= 94365717x + 776534	0.998	0.006	0.020	18%	85%
	DDD	0.01 - 0.13	y= 25634192x + 60787	0.993	0.011	0.037	-83%	82%
	DDT	0.01 - 0.13	y= 166281349x + 1600524	0.996	0.009	0.030	50%	84%
	Methoxychlor	0.01 - 0.13	y= 76145243x + 766864	0.992	0.012	0.040	86%	88%
OPPs	Dimethoate	0.01 - 0.13	y= 38512480x + 40798	0.996	0.010	0.033	36%	73%
	Chlorpyrifos-methyl	0.01 - 0.13	y= 38352460x + 141861	0.991	0.013	0.044	8%	70%
	Parathion-methyl	0.01 - 0.13	y= 37254725x + 117188	0.991	0.013	0.044	33%	77%
	Malathion	0.01 - 0.13	y=38867381x + 166301	0.997	0.009	0.030	12%	81%
	Chlorpyrifos	0.01 - 0.13	y= 35730243x + 112607	0.998	0.007	0.023	0%	76%

	Chlorfenvinphos	0.01 - 0.13	y= 20001864x + 26362	0.998	0.007	0.023	17%	83%
BFRs	TBECH	0.01 - 0.13	y = 14104536x + 909102	0.952	0.031	0.104	-9%	93%
	BDE 28	0.01 - 0.10	y= 81490036x + 858650	0.995	0.011	0.036	-20%	86%
	PBT	0.01 - 0.13	y= 64432907x + 343442	0.997	0.007	0.023	-10%	88%
	PBEB	0.01 - 0.13	y= 87862395x + 809774	0.995	0.011	0.036	-35%	87%
	BDE 47	0.01 - 0.13	y= 97516663x + 1702848	0.993	0.013	0.043	-70%	93%
	BDE 100	0.01 - 0.10	y= 103919127x + 376275	0.997	0.007	0.024	-58%	89%
	BDE 99	0.01 - 0.10	y= 98103175x + 466692	0.998	0.006	0.019	-36%	91%
	TBB	0.01 - 0.10	y= 70242770x + 1151459	0.998	0.007	0.022	-37%	91%
	BDE 153	0.01 - 0.10	y= 104926351x + 388905	0.997	0.007	0.022	-45%	90%
	BDE 154	0.01 - 0.10	y= 45652538x + 604600	0.993	0.010	0.034	-36%	86%
	BDE 183	0.01 - 0.10	y= 92242191x + 539132	0.999	0.004	0.013	-22%	88%
	BTBPE	0.01 - 0.13	y= 64760812x + 605681	0.992	0.012	0.042	25%	87%
PCBs	PCB 28	0.01 - 0.13	y= 102933267x + 1325126	0.998	0.007	0.023	-15%	62%
	PCB 52	0.01 - 0.13	y= 63106100x + 971630	0.992	0.012	0.040	-18%	76%
	PCB 101	0.01 - 0.13	y= 97975532x + 3013827	0.994	0.010	0.035	-20%	84%
	PCB 77	0.01 - 0.13	y= 101036917x + 754781	0.991	0.013	0.044	-41%	82%
	PCB 118	0.01 - 0.13	y= 126880256x + 1312831	0.996	0.009	0.029	-20%	82%
	PCB 114	0.01 - 0.13	y= 332139764x + 5533491	0.995	0.009	0.031	-32%	86%
	PCB 153	0.01 - 0.13	y= 136028816x + 1810527	0.995	0.010	0.033	-28%	86%
	PCB 138	0.01 - 0.10	y= 109538813x + 871840	0.999	0.002	0.008	-83%	81%
	PCB 126	0.01 - 0.13	y= 127729214x + 4143415	0.996	0.008	0.028	-45%	85%
	PCB 156	0.01 - 0.13	y= 261852734x + 3309590	0.998	0.006	0.021	-49%	87%
	PCB 157	0.01 - 0.13	y=228228539x + 2963534	0.992	0.012	0.041	-74%	87%
	PCB 180	0.01 - 0.13	y= 213945518x + 2986104	0.997	0.007	0.023	-56%	87%
	PCB 169	0.01 - 0.13	y= 150438200x + 2032588	0.992	0.013	0.042	-47%	95%
SMs	HHCB	0.01 - 0.13	y= 3018360x + 19270	0.994	0.013	0.045	93%	72%
	xylene	0.01 - 0.13	y= 638375x - 3424	0.992	0.013	0.042	90%	65%
	AHTN	0.01 - 0.10	y= 2589662x - 623	0.996	0.009	0.029	90%	68%
	Ketone	0.01 - 0.13	y= 14411585x - 2912	0.993	0.012	0.039	91%	77%

Table S4 - QuEChERS and clean-up optimization.

	Recoveries (50 µg/L)				
	QuEChERS			Clean-up graphitized carbon	
OCPs	Original	AOAC	EN	2 mg	5 mg
α-HCH	105%	**102%**	96%	**50%**	47%
HCB	84%	**85%**	75%	**37%**	28%
β-HCH	83%	**77%**	75%	**62%**	60%
Lindane	98%	**94%**	107%	**81%**	72%
δ-HCH	132%	**104%**	144%	**91%**	73%

Aldrin	89%	**85%**	79%	**58%**	54%
α-endosulfan	80%	**77%**	76%	**68%**	66%
DDE	96%	**89%**	83%	**68%**	66%
Dieldrin	102%	**97%**	93%	**70%**	70%
Endrin	129%	**127%**	122%	**70%**	68%
β-endosulfan	121%	**113%**	121%	**68%**	64%
DDD	111%	**105%**	107%	**74%**	75%
DDT	91%	**92%**	91%	**71%**	73%
Methoxychlor	142%	**131%**	131%	**75%**	69%

Table S5 - Validation parameters using shrimp matrix matched calibration for the selected OCPs, OPPs, BFRs, PCBs and SMs.

		Calibration range (ng/g ww)	Regression equation (ng/g ww)	Linearity ($R^{2)}$)	MDL (ng/g ww)	MQL (ng/g ww)	ME	Recoveries
OCPs	α-HCH	2.67 - 26.67	y= 26293x - 35928	0.997	1.52	5.07	-19%	96%
	HCB	2.67 - 26.67	y= 149710x + 57140	0.999	0.95	3.16	-100%	71%
	β-HCH	2.67 - 26.67	y= 7882x + 251167	0.997	1.54	5.15	-9%	83%
	Lindane	2.67 - 26.67	y= 23984x -22164	0.999	0.96	3.19	39%	91%
	δ-HCH	2.67 - 26.67	y= 18146x - 30396	0.996	1.91	6.35	-174%	56%
	Aldrin	2.67 - 26.67	y= 224426x + 108435	0.998	1.27	4.24	-135%	72%
	α-endosulfan	2.67 - 26.67	y= 59890x - 60884	0.997	1.56	5.21	47%	74%
	DDE	2.67 - 26.67	y= 50029x - 62724	0.997	1.54	5.13	57%	84%
	Dieldrin	2.67 - 26.67	y= 187368x - 80901	0.998	1.19	3.97	-212%	80%
	Endrin	2.67 - 26.67	y= 140881x - 64321	0.998	1.14	3.78	-95%	76%
	β-endosulfan	2.67 - 26.67	y= 44034x - 49111	0.998	1.39	4.63	11%	79%
	DDD	2.67 - 26.67	y= 38451x - 42109	0.998	1.43	4.77	35%	88%
	DDT	2.67 - 26.67	y= 56480x + 11441	0.995	2.03	6.78	14%	81%
	Methoxychlor	2.67 - 26.67	y= 16121x - 18222	0.991	2.89	9.63	-15%	83%
OPPs	Dimethoate	8.00 - 80.00	y= 23236x + 17097	0.995	6.39	21.31	29%	91%
	Chlorpyrifos-methyl	8.00 - 80.00	y= 20376x + 1149	0.998	4.23	14.10	6%	86%
	Parathion-methyl	8.00 - 80.00	y= 20453x - 10154	0.997	4.55	15.17	17%	60%
	Malathion	8.00 - 80.00	y= 19019x - 5561	0.997	4.51	15.02	7%	74%
	Chlorpyrifos	8.00 - 80.00	y= 15881x - 93	0.997	4.86	16.21	1%	86%
	Chlorfenvinphos	8.00 - 64.00	y= 7963x - 6454	0.994	5.79	19.31	12%	89%
BFRs	TBECH	4.00 - 40.00	y= 43101x + 140736	0.996	2.77	9.24	8%	91%
	BDE 28	4.00 - 40.00	y= 127797x + 316589	0.993	3.70	12.33	-29%	85%
	PBT	4.00 - 40.00	y= 94172x + 436079	0.996	2.84	9.46	-15%	65%
	PBEB	4.00 - 40.00	y= 108866x + 275105	0.997	2.41	8.04	-38%	66%
	BDE 47	4.00 - 40.00	y= 69674x + 34024	0.993	3.58	11.93	-174%	136%
	BDE 100	4.00 - 40.00	y= 81935x + 134040	0.998	1.87	6.22	-99%	87%

	BDE 99	4.00 - 40.00	y= 89632x + 522952	0.997	2.20	7.34	-72%	89%
	TBB	4.00 - 40.00	y= 49674x + 391723	0.998	1.89	6.32	-58%	86%
	BDE 153	4.00 - 40.00	y= 76402x + 151733	0.999	1.08	3.59	-92%	79%
	BDE 154	4.00 - 40.00	y= 84795x + 147501	0.998	2.03	6.76	-82%	80%
	BDE 183	4.00 - 40.00	y=72951x + 135649	0.999	1.28	4.27	-71%	76%
	BTBPE	4.00 - 40.00	y=51196x + 219525	0.998	1.91	6.38	3%	72%
PCBs	PCB 28	4.00 - 40.00	y= 104162x + 256379	0.996	2.62	8.75	-16%	80%
	PCB 52	4.00 - 40.00	y= 55416x + 227287	0.996	2.72	9.08	-26%	88%
	PCB 101	4.00 - 40.00	y= 80515x + 238679	0.997	2.53	8.44	-20%	82%
	PCB 77	4.00 - 40.00	y= 57279x + 233121	0.995	3.08	10.27	-34%	76%
	PCB 118	4.00 - 40.00	y= 70935x + 176582	0.997	2.60	8.65	-29%	76%
	PCB 114	4.00 - 40.00	y= 124222x + 350178	0.996	2.63	8.77	-29%	77%
	PCB 153	4.00 - 40.00	y= 87313x + 189294	0.997	2.31	7.71	-55%	82%
	PCB 138	4.00 - 40.00	y= 70795x + 208002	0.997	2.46	8.22	-93%	80%
	PCB 126	4.00 - 40.00	y= 70839x + 267188	0.997	2.52	8.40	-47%	73%
	PCB 156	4.00 - 40.00	y= 104655x + 176793	0.998	1.80	5.99	-86%	82%
	PCB 157	4.00 - 40.00	y= 81029x + 109421	0.998	1.88	6.27	-107%	84%
	PCB 180	4.00 - 40.00	y= 80140x + 271232	0.997	2.21	7.36	-87%	85%
	PCB 169	4.00 - 40.00	y= 50547x + 305253	0.997	2.44	8.14	-90%	82%
SMs	Celestolide	4.00 - 40.00	y= 579x - 817	0.999	1.48	4.94	38%	77%
	Ambrette	4.00 - 40.00	y= 154x + 355	0.995	3.36	11.21	55%	74%
	HHCB	4.00 - 40.00	y= 537x - 621	0.996	3.16	10.53	56%	79%
	MX	4.00 - 40.00	y= 129x + 666	0.994	3.41	11.38	58%	85%
	AHTN	4.00 - 40.00	y=522x + 651	0.998	2.18	7.28	45%	82%
	MK	4.00 - 40.00	y= 309x + 127	0.999	1.33	4.42	61%	94%

Table S6 - Reagents, solvents, and materials used in the extraction of the samples and in the analysis of the studied contaminants.

**Reagents, Solvents and materials**		**Supplier company**	**City**	**Country**
Acetonitrile	chromatographic grade	Merck	Darmstadt	Germany
Methanol	chromatographic grade	Scharlau	Barcelona	Spain
Ethyl acetate	Purity ≥ 99.8%	Merck	Darmstadt	Germany
Dichloromethane	chromatographic grade	Honeywell	Indiana	USA
n-Hexane	chromatographic grade	Merck	Darmstadt	Germany
D-(+)-Gluconic acid δ-lactone	Assay ≥ 99.0%	Sigma Aldrich	Darmstadt	Germany
QuEChERS extraction kit AOAC	6 g magnesium sulfate and 1.5 g sodium acetate	Agilent tecnologies	California	USA
QuEChERS extraction kit EN	1 g sodium chloride, 4 g magnesium sulfate, 1g sodium citrate and 0.5 g sodium hydrogencitrate sesquihydrate	Agilent tecnologies	California	USA
QuEChERS extraction kit original	1 g sodium chloride, 4 g magnesium	Agilent tecnologies	California	USA

Sodium sulfate anhydrous		Merk	Darmstadt	Germany
Graphitized carbon black		Agilent tecnologies	California	USA
Dispersive solid phase AOAC clean up	150 mg anhydrous magnesium sulphate, 50 mg PSA and 50 mg C18	Agilent tecnologies	California	USA
Hydrochloric acid (HCl)	37%	Carlo Erba	Rodano	Italy
Deionized water	Resistivity of 15.0 MΩ.cm<br>Produced in a Elix apparatus	Milipore	Molsheim	France
Ultrapure water	Resistivity of 18.2 MΩ.cm<br>Produced using a Simplicity 185 system	Millipore	Molsheim	France
Strata C18-E (500 mg, 3 mL)		Phenomenex	California	USA

Table S7 - Physico-chemical characteristics, molecular mass, and the supplier company of the studied contaminants.

	Contaminants		Log Kow	CAS	Molecular weight (g/mol)	Supplier company
OCPs	α- hexachlorocyclohexane	α-HCH	3.80 [a]	319-84-6	290.8	Sigma Aldrich (Darmstadt, Germany)
	β- hexachlorocyclohexane	β-HCH	3.78 [a]	118-74-1	284.8	
	δ- hexachlorocyclohexane	δ-HCH	4.14 [a]	319-85-7	290.8	
	lindane		3.72 [a]	58-89-9	290.8	
	hexachlorobenzene	HCB	5.73 [b]	319-86-8	290.8	
	1,1,1 trichloro-2,2- bis-(p-chlorophenyl) ethane)	*o,p′*-DDT	6.91 [b]	309-00-2	364.9	
	2,2-bis(p-chlorophenyl)-1,1-dichloroethylene	*p,p'*-DDE	6.51 [b]	959-98-8	406.9	
	dichlodiphenyldichloro-ethane	*p,p'*-DDD	6.02 [b]	72-55-9	318.0	
	aldrin		6.50 [b]	60-57-1	380.9	
	dieldrin		5.40 [b]	72-20-8	380.9	
	endrin		5.20 [b]	33213-65-9	406.9	
	α- endosulfan		3.83 [b]	72-54-8	320.0	
	β- endosulfan		3.62 [b]	50-29-3	354.5	
	methoxychlor		5.08 [b]	72-43-5	345.7	
OPPs	dimethoate		0.78 [b]	60-51-5	229.3	Sigma Aldrich (Darmstadt, Germany)
	chlorpyrifos-methyl		4.31 [b]	5598-13-0	322.5	
	parathion-methyl		2.86 [b]	298-00-0	263.2	
	malathion		2.36 [b]	121-75-5	330.4	
	chlorpyrifos		4.96 [b]	2921-88-2	350.6	
	chlorfenvinphos		3.81 [b]	470-90-6	359.6	
BFRs	2,4,4′-tribromodiphenyl ether	BDE 28	5.94 [c]	41318-75-6	406.89	Isostandards Material, S.L. (Madrid, Spain)
	2,2′,4,4′- tetrabromodiphenyl ether	BDE 47	6.81 [c]	41318-75-6	405.8	
	2,2′,4,4′,5-pentabromodiphenyl ether	BDE 99	7.32 [c]	87-83-2	485.6	
	2,2′,4,4′,6-pentabromodiphenyl ether	BDE 100	7.24 [c]	85-22-3	499.6	
	2,2′,4,4′,5,5′-hexabromodiphenyl ether	BDE 153	7.90 [c]	59080-40-9	627.6	

	2,2′,4,4′,5,6′-hexabromodiphenyl ether	BDE 154	7.82 [c]	189084-64-8	563.6	
	2,2′,3,4,4′,5′,6-heptabromodiphenyl ether	BDE 183	8.27 [c]	32534-81-9	563.6	
	1,2-dibromo-4-(1,2-dibromoethyl)-cyclohexane	TBECH	4.82 [d]	3322-93-8	427.80	
	pentabromotoluene	PBT	6.25 [d]	68631-49-2	643.5	
	pentabromoethylbenzene	PBEB	6.76 [d]	207122-15-4	643.5	
	2-ethylhexyl 2,3,4,5-tetrabromobenzoate	TBB	7.73 [d]	207122-16-5	721.4	
	1,2-bis(2,4,6-tribromophenoxy) ethane	BTBPE	8.31 [d]	37853-59-1	687.6	
PCBs	2,4,4′-Trichlorobiphenyl	PCB 28	5.67 [e]	7012-37-5	257.5	Techno Spec S.L. (Barcelona, Spain)
	2,2′,5,5′-Tetrachlorobiphenyl	PCB 52	5.84 [e]	35693-99-3	292.0	
	2,2′,4,5,5′-Pentachlorobiphenyl	PCB101	6.38 [e]	37680-73-2	326.4	
	3,3′,4,4′-Tetrachlorobiphenyl	PCB 77	6.36 [e]	32598-13-3	292.0	
	2,3′,4,4′,5′-Pentachlorobiphenyl	PCB 118	6.74 [e]	31508-00-6	326.4	
	2,3,4,5,4'-Pentachlorobiphenyl	PCB 114	6.65 [e]	74472-37-0	326.4	
	2,2′,4,4′,5,5′-Hexachlorobiphenyl	PCB 153	6.92 [e]	35065-27-1	360.9	
	2,2′,3,4,4′,5′-Hexachlorobiphenyl	PCB 138	6.83 [e]	35065-28-2	360.9	
	3,3',4,4',5'-Pentachlorobiphenyl	PCB 126	6.89 [e]	57465-28-8	326.4	
	2,3,3′,4,4′,5-Hexachlorobiphenyl	PCB 156	7.18 [e]	38380-08-4	360.9	
	2,3,3',4,4',5'-Hexachlorobiphenyl	PCB 157	7.18 [e]	69782-90-7	360.9	
	2,2′,3,4,4′,5,5′-Heptachlorobiphenyl	PCB 180	7.36 [e]	35065-29-3	395.3	
	3,4,5,3',4',5'-Hexachlorobiphenyl	PCB 169	7.42 [e]	32774-16-6	360.9	
SMs	galaxolide	HHCB	5.90 [b]	13171-00-1	244	LGC Standards (Middlesex, UK)
	tonalide	AHTN	5.70 [b]	83-66-9	268	
	ketone		4.30 [b]	12222-05-5	258	
	xylene		3.12 [b]	81-15-2	297	
	celestolide		5.70 [b]	21145-77-7	258	
	ambrette		4.00 [b]	81-14-1	294	
IS	4.4'- dichlorobenzophenone			90-98-2	251.1	Sigma Aldrich (Darmstadt, Germany)
	triphenylphosphate			115-86-6	326.3	
	5′- fluoro-2,3′,4,4′,5-pentabromodiphenyl ether			446254-80-4	564.7	Isostandards Material, S.L. (Madrid, Spain)
	AHTNd3			1396967-82-0	261	LGC Standards (Middlesex, UK)

[a] National Toxicology Program, 2011. Hexachlorocyclohexane (technical grade), and other hexachlorocyclohexane isomers. Rep. Carcinog. 12, 256–258

[b] Hazardous Substances Data Bank (HSDB)

[c] Braekevelt, E. et al., 2003. Direct measurement of octanol-water partition coefficients of some environmentally relevant brominated diphenyl ether congeners. Chemosphere. 51, 563-567

[d] Dong, L. et al., 2021. New understanding of novel brominated flame retardants (NBFRs): Neuro(endocrine) toxicity. Ecotoxicology and Environmental Safety. 208, 111570

[e] IARC, 2016. Polychlorinated biphenyls. IARC monographs on the evaluation of carcinogenic risks to humans. 107, 9-500.

Table S8 - GC-MS and MS/MS conditions for SMs analysis.

SMs	CAS	Molecular weight (g/mol)	RT (min)	Quantifier ion (m/z)*	Qualifier ions (m/Z)*	MRM transitions (m/z)*
Celestolide	13171-00-1	244	14.83	229	173, 244	229 → 173
Ambrette	83-66-9	268	20.09	253	91, 223	253 → 91
HHCB	12222-05-5	258	20.74	243	213, 258	243 → 213
Xylene	81-15-2	297	21.29	282	265, 297	282 → 265
AHTN	21145-77-7	258	21.34	243	187, 258	243 → 187
Ketone	81-14-1	294	26.35	279	191, 294	279 → 191
AHTN-d3 (IS)	1396967-82-0	261	21.23	246	160, 190	n.a

n.a. - not applicable; * Dong et al., 2014 and Kuklenyik et al., 2007.

Table S9 - GC-MS/MS conditions for OCPs, OPPs, BFRs, PCBs analysis.

	Compounds	EDC or FPD<br>RT (min)	MS/MS<br>MRM transitions (m/z)*
OCPs	α-HCH	16.41	183 → 179
	HCB	16.67	284 → 214
	β-HCH	17.18	183 → 179
	Lindane	17.34	183 → 179
	δ-HCH	17.99	183 → 179
	Aldrin	20.12	263 → 191
	α-endosulfan	21.90	195 → 170
	DDE	22.35	318 → 281
	Dieldrin	22.49	243 → 211
	Endrin	22.98	245 → 173
	β-endosulfan	23.17	195 → 170
	DDD	23.27	235 → 165
	DDT	23.36	235 → 165
	Methoxychlor	25.19	227 → 197
OPPs	Dimethoate	10.15	125 → 62
	Chlorpyrifos-methyl	13.98	286 → 241
	Parathion-methyl	14.12	263 → 246
	Malathion	16.76	173 → 99
	Chlorpyrifos	17.13	314 → 258
	Chlorfenvinphos	20.21	267 → 159
BFRs	TBECH	22.07	267 → 79
	BDE 28	28.11	408 → 248
	PBT	28.43	486 → 326
	PBEB	29.92	500 → 406
	BDE 47	36.72	486 → 326
	BDE 100	43.76	564 → 404
	BDE 99	45.80	564 → 404

	TBB	46.35	421 → 393
	BDE 153	51.59	644 → 484
	BDE 154	54.85	644 → 484
	BDE 183	67.00	720 → 562
	BTBPE	71.65	359 → 278
PCBs	PCB 28	19.42	256 → 186
	PCB 52	20.64	292 → 255
	PCB 101	24.52	326 → 256
	PCB 77	26.52	292 → 222
	PCB 118	28.03	326 → 256
	PCB 114	28.70	326 → 256
	PCB 153	29.45	360 → 290
	PCB 138	31.40	360 → 290
	PCB 126	32.07	326 → 256
	PCB 156	35.24	360 → 290
	PCB 157	35.67	360 → 290
	PCB 180	36.56	394 → 324
	PCB 169	38.18	360 → 290

* Fernandes et al., 2011; Sapozhnikova, 2018 and Sapozhnikova and Lehotay, 2013.
